# Evaluation of the 2007 WHO guideline to diagnose smear negative tuberculosis in an urban hospital in Ethiopia

**DOI:** 10.1186/1471-2334-13-427

**Published:** 2013-09-11

**Authors:** Gemeda Abebe, Amare Deribew, Ludwig Apers, Alemseged Abdissa, Yibeltal Kiflie, Olivier Koole, Robert Colebunders

**Affiliations:** 1 Department of Medical Laboratory Sciences and Pathology, Jimma University, Jimma, Ethiopia; 2 International Medical Corps, Khartoum, Sudan; 3 Department of Clinical Sciences, Institute of Tropical Medicine, Antwerp, Belgium; 4 Department of Health Service Management, Jimma University, Jimma, Ethiopia; 5 Department of Epidemiology and Social Medicine, University of Antwerp, Antwerp, Belgium

**Keywords:** Smear negative, WHO, Tuberculosis, HIV, Diagnosis, Tuberculosis

## Abstract

**Background:**

The 2007 World Health Organization (WHO) guideline to diagnose smear-negative tuberculosis (TB) in HIV-prevalent settings was mainly based on expert advice and therefore requires evaluation in real life situations.

**Methods:**

In 2009, this guideline was introduced at the ALERT hospital in Ethiopia. From October 2009 to January 2011, the accuracy of the guideline was evaluated using *Mycobacterium tuberculosis* culture positivity as reference standard in HIV positive TB suspects.

**Results:**

A total of 459 TB suspects were enrolled during the study period; 336 (73.2%) were HIV positive. Acid fast bacilli sputum smear microscopy was done for 74.7% (251/336) HIV positive TB suspects; 94.4% (237/251) were smear negative. A chest X-ray was performed in 92.8% (220/237) and a *Mycobacterium tuberculosis* culture in 63.7% (151/237). The median TB diagnostic delay for smear negative cases was 3 days (interquartile range 3–4 days). Of the 75 patients diagnosed with smear negative pulmonary TB, 89. 4% (67/75) were diagnosed by chest X-ray, 9.4% (7/75) by culture and 1.3% (1/75) by clinical suspicion only. In 147 smear negative TB suspects *Mycobacterium tuberculosis* culture and chest X-ray results were available. Among these 147 patients, the sensitivity of the chest X-ray to diagnose smear negative TB in HIV-positive TB suspects was 53.3% (95% CI: 26.7-78.7); the specificity 67.4% (95% CI: 58.7-75.3).

**Conclusion:**

The 2007 WHO diagnostic algorithm for the diagnosis of smear negative TB is likely to reduce the diagnostic delay and therefore decrease morbidity and mortality of TB in a HIV prevalent settings like Ethiopia.

## Background

The WHO has classified Ethiopia among the 22 high burden countries with TB. In 2011, it was estimated that in Ethiopia the annual TB incidence was 258/100,000, the prevalence of all forms of TB 237/100,000 and the TB mortality rate 18/100,000 [1].

In 2011 the adult HIV prevalence in Ethiopia was 1.5% [2] and the prevalence of HIV in newly diagnosed patients with TB 17% [1]. TB and HIV co-infection presents special diagnostic and therapeutic challenges and constitutes an immense burden on healthcare systems of countries with high prevalence [3-5]. In patients with HIV, especially patients with low CD4 T cell counts, TB is more difficult to diagnose because they often present with smear negative pulmonary TB and extrapulmonary TB [6]. This contributes to low case detection rates [7] and TB diagnosis delay. Therefore, in September 2005, WHO convened an expert group to review the 2003 WHO recommendations concerning the diagnosis of smear-negative TB in HIV-prevalent settings.

Major changes were proposed. In 2007 a new diagnostic algorithm was endorsed. The new recommendations included: all TB suspects need to be tested for HIV, in HIV positive individuals only two sputa (spot-morning) need to be obtained and one positive smear is enough to diagnose TB; a chest X-ray and *Mycobacterium tuberculosis* cultures should be performed if smears are negative and an antibiotic trial should not be done as a diagnostic test [8].

In Ethiopia, the new WHO diagnostic algorithm was adopted by the end of 2009. The adult HIV prevalence in Addis Ababa during the 2010 was 5.2% [2]. The objective of this study was to evaluate the operational performance of the 2007 WHO diagnostic algorithm to diagnose smear negative TB in HIV-positive patients in a tertiary referral hospital in Addis Ababa.

## Methods

### Setting and design

From October 2009 to January 2011, we evaluated the operational performance of the WHO 2007 smear negative TB diagnostic algorithm at the ALERT hospital. The ALERT hospital is located in Addis Ababa. It is equipped with a TB culture facility. Prior to the implementation of the study, all health professionals (doctors, nurses, laboratory and X-ray technicians) involved in TB/HIV services were trained during one week about the 2007 WHO guideline. One local physician was appointed to supervise the research activities. Monthly supervisions to the hospital were done by the investigators.

TB suspects, 15 years or older, were enrolled. Patients were considered TB suspects according to WHO criteria [8] if they presented with cough for at least 2 weeks or weight loss with night sweats or feeling feverish with T>37.5°C, breathlessness due to pleural effusion or pericarditis or enlarged (>2 cm) glands in neck or armpits. Patients with danger signs (defined by WHO [8]) were excluded.

In TB suspects with enlarged peripheral lymph nodes fine needle aspiration (FNA) was performed using a 22-gauge needle attached to a 10-cc syringe. Aspirates were macroscopically evaluated for caseation and smears were air dried, stained with Wright stain and examined cytologically by a pathologist. Cytology was considered to be diagnostic for TB in the presence of epitheloid cell granulomas and caseous necrosis with or without Langhans giant cells [9,10].

Sputum samples from TB suspects that were negative by AFB microscopy were cultured for the presence of *Mycobacterium tuberculosis* complex by Löwenstein-Jensen (LJ) slants (but only one culture per patient was performed). The samples were processed by the standard N-acetyl L-cysteine (NALC)-NaOH method [11] and concentrated at 3000 g for 15 minutes. Sediments were reconstituted to 2.5 ml with phosphate buffer pH 6.8, to make the inoculums for the cultures. Two Lowenstein-Jensen slants, one containing 0.75% glycerol and the other containing 0.6% pyruvate, were inoculated with the sediment and incubated at 37°C. Cultures were considered negative when no colonies were seen after 8 weeks of incubation.

The following TB case definitions were used as indicated in the WHO 2007 guideline [8]. 1) Smear-positive pulmonary tuberculosis*-* One sputum smear examination positive for acid-fast bacilli (AFB) AND- laboratory confirmation of HIV infection OR strong clinical evidence of HIV infection. 2) Smear-negative pulmonary tuberculosis- At least two sputum specimens negative for AFB AND radiographical abnormalities consistent with active tuberculosis AND- laboratory confirmation of HIV infection OR strong clinical evidence of HIV infection AND decision by a clinician to treat with a full course of antituberculosis chemotherapy OR a patient with AFB smear-negative sputum which is culture-positive for *Mycobacterium tuberculosis*. 3) Extrapulmonary tuberculosis- One specimen from an extrapulmonary site culture-positive for *Mycobacterium tuberculosis* or smear-positive for AFB OR histological or strong clinical evidence consistent with active extrapulmonary tuberculosis AND- laboratory confirmation of HIV infection OR strong clinical evidence of HIV infection AND a decision by a clinician to treat with a full course of antituberculosis chemotherapy.

All consenting TB suspects were offered HIV testing and counseling. HIV testing was done according to the Ethiopian national guidelines. HIV screening was performed using the KHB test (Shanghai Kehua Bio-Engineering Ltd, Shanghai, China; 2008). A positive sample was retested using the STAT-PAK test (Chembio Diagnostic System Inc, Medford, NY, USA; 2008). Uni-Gold (Trinity Biotech PLC, Ireland) was used as a tiebreaker.

### Data analysis

Data were double entered using EpidData software version 3.1(EpidData, Norway, 2006). For analysis, the data were exported to SPSS version 15.0 statistical software (SPSS Inc. Chicago, 2007). Descriptive analysis was done to depict the TB suspects’ characteristics. The incremental yield of the different investigations was defined as the proportion of patients additionally diagnosed by X-ray if smear-negative, by clinical suspicion in smear-negatives without an X-ray abnormality consistent with active TB, and ultimately by culture if all of the above were negative. Health system diagnostic delay (time between first presentation at the clinic and date of diagnosis) was calculated for all patients. Sensitivity and specificity of the chest X-ray was calculated using *Mycobacterium tuberculosis* culture positivity as reference standard. Patients without X-ray or culture results were excluded from this analysis. Confidence intervals around sensitivity and specificity were calculated using the Wilson’s method.

### Ethical considerations

The study protocol was approved in Ethiopia by the ethical review committees of Jimma University, Armauer Hansen Research Institute and Ministry of Science and Technology. In Belgium it was approved by the ethical review committees of the Institute of Tropical Medicine and the University hospital of Antwerp. Written consent was obtained from the study participants.

## Results

### Patient characteristics

A total of 459 TB suspects were screened. The median age of the TB suspects was 35 years (interquartile range 30–45 years); 55.1% (253/459) were females. The majority of the TB suspects were urban residents (98.3% (451/459), had cough since at least 2 weeks (91.1% (418/459)) and were HIV positive (73.2% (336/459)). Among the HIV positive TB suspects 88.7% (298/336) had cough since at least 2 weeks. Other symptoms reported included weight loss (56.0% (188/336)), fever (58.0% (195/336)), shortness of breath (30.4% (102/336)) and lymph node swelling (13.4% (45/336)) (Table 1).

**Table 1:** TB suspects characteristics by HIV status

**Characteristics**	**All TB suspects**	**HIV positive TB suspects**	**P-value**
**TB suspects, n**	459 (100.0)	336 (73.2)	<0.001
**Age in years, Median (IQR)**	35 (30.0–45.0)	35 (29.3–42.0)	
**Female sex, n (%)**	253 (55.1)	194 (57.7)	0.06
**Urban resident, n (%)**	451 (98.3)	334 (99.4)	0.02
**Symptoms**			
Cough for 2 weeks or more, n (%)	418 (91.1)	298 (88.7)	0.03
Weight loss, n (%)	236 (51.4)	188 (56.0)	0.001
Fever, n (%)	293 (63.8)	195 (58.0)	<0.01
Current shortness of breath, n (%)	168 (36.5)	102 (30.4)	<0.001
Lymph node swelling, n (%)	49 (10.7)	45 (13.4)	0.002

### Operational performance

#### Diagnostic workup

**Figure 1:**
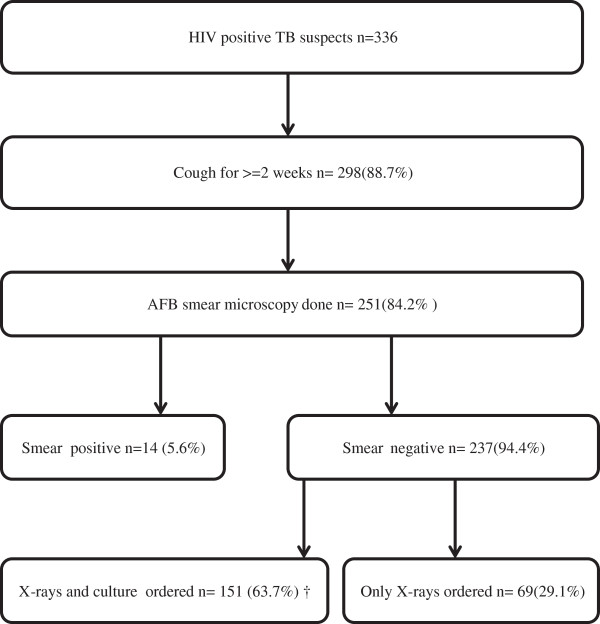
**Flow of the diagnostic process in HIV positive TB suspects at the ALERT hospital, Addis Ababa, Ethiopia.** † four culture results were not available (2 contaminated and 2 not done).

Sputum AFB microscopy was done for 84.2% (251/298) of the HIV positive TB suspects with cough of two weeks or more. 94.4% (237/251) were sputum AFB smear negative. A chest X-ray was performed in 92.8% (220/237) and a *Mycobacterium tuberculosis* culture was ordered in 63.7% (151/237) of the smear negative TB suspects. A chest X-ray was performed in all TB suspects with a *Mycobacterium tuberculosis* culture. Four culture results were not available (2 contaminated, 2 not done) (Figure 1).

### Diagnostic outcomes of TB suspects and diagnostic delay

Of the 336 HIV positive TB suspects, 36.6% (123/336) were diagnosed with TB: 11.4% (14/123) smear positive pulmonary TB, 61.0% (75/123) smear negative pulmonary TB, 19.5% (24/123) TB lymphadenitis and 8.1% (10/123) other forms of TB. *Mycobacterium tuberculosis* culture was positive in 10.2% (15/147) and a chest X-ray result was consistent with active TB in 35.0% (77/220) of the smear negative TB suspects. The median diagnostic delay for smear negative pulmonary TB cases was 3 days (interquartile range 3–4 days). The median number of visits before the diagnosis of smear negative TB was 2 (Table 2).

**Table 2:** Diagnostic outcomes of HIV positive TB suspects (n=336)

**Outcome**	**N(%)**
**Diagnosed with TB**	123 (36.6)
**Type of TB (n=123)**	
Smear Positive pulmonary TB	14 (11.4)
Smear Negative Pulmonary TB	75 (61.0)
TB Lymphadenitis	24 (19.5)
Other forms of TB	10 (8.1)
**Positive culture result (n=147)#**	15 (10.2)
**X-ray result consistent with active TB (n=220)#**	77 (35.0)
**WHO stage for co-infected patients**	
III	89 (72.4)
IV	34 (27.6)
**Median diagnostic delay in days (IQR)**	3 (3–4)
**Median number of visits before smear negative TB diagnosis**	2

#Smear negative TB suspects, *IQR* interquartile range.

### Incremental yield of the different investigations

Of the 123 patients diagnosed with TB, 11.4% (14/123) were diagnosed by AFB smear microscopy, 18.7% (23/123) by FNA cytology, 62.6% (77/123) by chest X-ray, 5.7% (7/123) by culture and 1.6% (2/123) by clinical suspicion only. Of the 75 patients diagnosed with smear negative pulmonary TB, 89. 4% (67/75) were diagnosed by chest X-ray, 9.4% (7/75) by culture and 1.3% (1/75) by clinical suspicion only (Table 3).

**Table 3:** Incremental yield of the 2007 guideline in HIV patients with all forms of TB and smear negative pulmonary TB

**Method**	**All patients with TB-HIV co infection (n=123)**	**HIV patients with smear negative pulmonary TB (n=75)**
AFB sputum smear microscopy	14 (11.4%)	NA
FNA cytology	+23 (18.7%)	NA
Clinical suspicion	+2 (1.6%)	+1 (1.3%)
Chest X- ray	+77 (62.6%)	+67 (89.3%)
Culture	+7 (5.7%)	+7 (9.4%)

*NA* Not applicable.

### Diagnostic accuracy of the chest X-ray to diagnose smear negative TB

Of the HIV positive smear negative TB suspects, in 151 a *Mycobacterium tuberculosis* culture was ordered and in 147 a culture result obtained (Table 4). Among these 147 patients the sensitivity of the chest X-ray was 53.3% (95% CI: 27.7-78.7) and the specificity 67.4% (95% CI: 58.7-75.3). The positive predictive value was 15.7% (95% CI: 7.1-28.6) and the negative predictive value 92.7% (95% CI: 85.6-97.0).

**Table 4:** Comparison of the chest X-ray to diagnose smear negative TB with culture in HIV positive patients

**Chest X-ray**	**Culture result**	
	**Positive**	**Negative**
Consistent with TB	8	43
Not consistent with TB	7	89

## Discussion

Since the WHO 2007 guideline for the diagnosis of smear negative TB was largely based on expert opinion, validation was required. A randomized controlled trial comparing the WHO 2003 with the 2007 guidelines was considered to be unethical. Therefore we were only able to evaluate the 2007 guidelines during its implementation in an urban reference hospital in Ethiopia.

Our study suggests that the 2007 diagnostic algorithm may reduce the health system related diagnostic delay of smear negative TB. Indeed, before the introduction of the new guideline Demissie et al. reported a diagnostic delay of 6 days in all public health centers in Addis Ababa [12]. Despite the fact that the latter study was done in other health centers in Addis Ababa and did not report separately for HIV positive TB patients, the difference is striking (only 3 days in our study). At the Nekemte referral hospital in the west part of Ethiopia, which was still implementing the 2003 guideline during our study period, the median diagnostic delay for smear negative TB cases was even 14 days (Gemeda Abebe, personal communication). Delay in TB diagnosis is an important contributor of excess morbidity and mortality in HIV-infected people [13,14]. Delay in TB diagnosis is particularly problematic in HIV-infected individuals because in those patients TB presents more commonly as a disseminated disease [15] and disease progression is more rapid [16]. In our study the median number of visits by patients before a diagnosis of smear negative TB diagnosis was made was 2. According to the WHO 2007 guideline the total number of visits should not be more than 4 [8].

A high number of smear negative TB cases (89.3% of smear negative pulmonary TB cases) were diagnosed on the basis of X-ray results. This is similar to a study in Cambodia where 50.5% of the HIV positive TB cases were diagnosed on the basis of radiology [17]. The diagnostic sensitivity of the chest X-ray in our study was 53.3% (95% CI: 27.7-78.7). This result is comparable with reports from India (72%) [18], Kenya (55%) [19] and Uganda [20]. The specificity of 67.4% in our study is similar to the report from Kenya (72.9%) [19] but higher than the report from Uganda (22%) [20]. The chest X-ray had excellent negative predictive value but poor positive predictive value. Culture contributed to an additional diagnosis of only 7 cases (11.9%) of the smear negative TB. It is evident that we need diagnostic tools with a shorter turnaround time than culture. In a study in South Africa the GeneXpert MTB/RIF assay increased the case detection rate by 45% [21]. However, in resource limited settings like Ethiopia where GeneXpert MTB/RIF is only available in a few research sites, the 2007 WHO guideline for the diagnosis of smear negative TB is still relevant. FNA cytology contributed to the diagnosis of an additional 23 (18.7%) TB cases.

Our study has several limitations. Firstly, it was a single center based study; as a result the performance of the guideline at different levels of HIV and TB prevalence could not be evaluated. Secondly, we were unable to directly compare the performance of the new with the old WHO guidelines. Thirdly, due to the fact that not all TB suspects with cough were able to produce sputum with required quantity and quality, in an appreciable number of TB suspects no AFB or culture results were obtained. Finally, the solid culture used as reference standard for detecting TB may have under estimated the culture positivity rate. Culturing multiple specimens on liquid media would have detected more TB cases [22-24] and may have influenced the accuracy of chest X-ray. The value of our study lies in the description of the operational benefits of the new guidelines in a real life situation in a high HIV prevalent setting.

## Conclusion

The 2007 WHO diagnostic algorithm for the diagnosis of smear negative TB is likely to reduce the diagnostic delay and therefore decrease morbidity and mortality of TB in a HIV prevalent settings like Ethiopia.

## Competing interests

The authors declare that there is no competing interest.

## Authors’ contributions

GA was involved in the conception and design of the study, coordinated the field work, analyzed the data and drafted the manuscript. AD was involved in the conception, design of the study, field work and review of the article. LA was involved in the conception, design and reviewed the article. AA and YK participated in the design, field work and reviewed the article. OK reviewed the article. RC participated in the conception, design, and critically reviewed the article. All authors read and approved the final manuscript.

## Pre-publication history

The pre-publication history for this paper can be accessed here:

http://www.biomedcentral.com/1471-2334/13/427/prepub
